# Monoclonal Antibody against G Glycoprotein Increases Respiratory Syncytial Virus Clearance In Vivo and Prevents Vaccine-Enhanced Diseases

**DOI:** 10.1371/journal.pone.0169139

**Published:** 2017-01-11

**Authors:** Hyo-Jeong Lee, Jeong-Yoon Lee, Min-Hee Park, Joo-Young Kim, Jun Chang

**Affiliations:** Graduate School of Pharmaceutical Sciences, Ewha Womans University, Seoul, Korea; University of Iowa, UNITED STATES

## Abstract

Respiratory syncytial virus (RSV) is a common cause of lower respiratory tract illness in infants, young children, and the elderly. The G glycoprotein plays a role in host cell attachment and also modulates the host immune response, thereby inducing disease pathogenesis. We generated two monoclonal antibodies (mAbs; 5H6 and 3A5) against G protein core fragment (Gcf), which consisted of amino acid residues 131 to 230 from RSV A2 G protein. Epitope mapping study revealed that 5H6 specifically binds to the G/164-176 peptide that includes conserved sequences shared by both RSV A and B subtypes, and 3A5 binds to the G/190-204 peptide. Studies with mutant Gcf proteins in which cysteine residues were substituted with alanine revealed that 5H6 requires four cysteines for binding and 3A5 binds to Gcf variants with alanine substitutions better than wild-type. To determine if these mAbs reduce pulmonary viral infection, BALB/c mice were administered mAb and subsequently challenged with RSV. On day 4 post-infection, lung viral titers were reduced by up to 93% with the 5H6 injection and 90% with the 3A5 injection, indicating that prophylactic injection of these mAbs contributes to RSV clearance *in vivo*. Importantly, 5H6 injection reduced vaccine-enhanced diseases. Overall, our results suggest that this novel anti-G mAb could be used as a prophylactic regimen against RSV diseases.

## Introduction

Respiratory syncytial virus (RSV), a orthopneumovirus of the *Pneumoviridae* family, causes lower respiratory tract illness including bronchiolitis, pneumonia, and asthma in infants, young children, and the elderly [1, 2]. Many infants are infected with RSV at least once in their first two years of life. Common symptoms caused by RSV include cough, fever, wheezing, and rhinorrhea [3]. Globally, 3.4 million people were hospitalized and 66,000–199,000 people died due to severe symptoms [4–6]. In the 1960s, a formalin-inactivated RSV (FI-RSV) as a vaccine candidate was tested in clinical trials. However, FI-RSV trial resulted in the death of two toddlers, and most of the volunteers were hospitalized due to vaccine-enhanced disease after subsequent natural RSV infection [7–10]. Despite prolonged efforts to develop vaccines, there is no licensed vaccine to prevent RSV infection yet. Currently, prophylaxis with a humanized monoclonal antibody (mAb), Palivizumab, against the F protein has been shown to be effective in preventing viral infection [11, 12].

Among three surface proteins of RSV (F, G, and SH), the G glycoprotein plays a role in host cell attachment and interacts with glycosaminoglycans, CX3CR1, L-selectin-like molecules, L-SIGN, and DC-SIGN on the cell surface [13–16]. The G protein has a central conserved domain that spans amino acids 155 to 206 and includes 13 amino acids (positions 164–176 in strain A2) shared by both RSV A and B subtypes [17, 18]. The G protein forms disulfide bonds between four cysteines (between Cys-173 and Cys-186 and between Cys-176 and Cys-182) in the central conserved domain [19]. Conserved cysteine residues are necessary for induction of immune responses against RSV [20]. The G protein also modulates the host immune response via the CX3C motif that mimics fractalkine/CX3CL1. The G protein containing CX3C motif binds to the CX3CR1 receptor and has leukocyte chemoattractant activity, thereby inducing disease pathogenesis [15, 21, 22]. It has been shown that the G protein modulates CX3CR1+ T-cell trafficking to the lungs, and downregulates the Th1-mediated immune responses and enhances Th2-biased immune responses, because CX3CR1 is generally expressed by Th1-polarized cells [23–25].

We have been previously shown that intranasal immunization of G protein core fragment (Gcf), which consists of amino acids 131 to 230 from RSV A2 G proteins, induces strong serum IgG responses and provides protection against RSV challenge [20]. So, we hypothesized that anti-Gcf mAbs might be useful for prophylaxis and reduction of disease pathogenesis. To this end, we generated monoclonal antibodies against Gcf and investigated the epitope and binding characteristics, neutralization activity in vitro and in vivo, and prophylactic effects on vaccine-enhanced diseases.

## Materials and Methods

### Monoclonal antibody preparation

To generate mAbs against Gcf, mice were injected with Gcf. After the B cells were isolated from the spleen, they were fused with immortalized myeloma cells. For selection of hybridomas that produce specific antibodies against Gcf, ELISA was performed using hybridoma culture supernatants. Based on the affinity measurement, two mAb clones (5H6 and 3A5) were selected and purified for analysis.

### Cell and virus preparations

The HEp-2 cells (ATCC, Manassas, VA) were grown in MEM (Life Technologies, Gaithersburg, MD) supplemented with 10% heat-inactivated FBS, 2 mM glutamine, 20 mM HEPES, nonessential amino acids, penicillin, and streptomycin. The RSV A2 was propagated in HEp-2 cells and stocks were prepared as described previously [20]. RSV A2 titer was determined by standard plaque assay.

### Plasmid construction and Gcf purification

The expression plasmid encoding Gcf is described elsewhere [20]. The mutant Gcf in which four cysteine residues (Cys-172, Cys-176, Cys-182, Cys-186) were substituted with alanine was generated by mega-PCR with mutagenic primers [26]. The constructed plasmid was transformed into competent *E*.*coli* BL21(DE3) cells (Novagen), and overexpression and purification of Gcf and its derivatives was performed as described previously [20]. To remove endotoxins, proteins were treated with 1% Triton X-114 as previously described [27]. The endotoxin levels in the protein were measured by the limulus amebocyte lysate (LAL) assay kit, according to the manufacturer’s instructions (Lonza, Switzerland). The endotoxin level was less than 5 EU/mg. For further purification, proteins were loaded onto the Superdex-75 column (GE Healthcare) after equilibration with PBS. Proteins were stored in aliquots at –80°C until use.

### ELISA

Biotinylated peptides that corresponded with G protein B cell epitopes were synthesized for epitope mapping (peptide 144–159, 164–176, 174–187, 190–204) [28]. Then, 96-well MaxiSorp plates (Nunc, Roskilde, Denmark) were coated overnight with 200 ng of streptavidin in PBS per well. The plates were washed with PBST (0.05% Tween 20 in PBS), and 200 ng of the biotinylated peptide were added to each well. For culture supernatant as coating antigens, HEp-2 cells were infected with RSV A2 at an MOI of 0.01 or recombinant adenovirus expressing tandem repeats of copies of Gcf (rAd/3XG) at an MOI of 100, and 48 hr later, the serum-free media were harvested and clarified. 96-well plates were coated with 5 μg protein of culture supernatants. For antigens expressed in *E*.*coli*, 96-well plates were coated overnight with 50 ng of Gcf and mutant Gcf in PBS. After antigen-coated plates were blocked with PBS containing 1% skim milk, two-fold dilutions of 5H6 and 3A5 in PBST-containing 1% skim milk were added. After incubation with horseradish peroxidase (HRP)-conjugated rabbit anti-mouse IgG (Abcam, Cambridge, UK), the plates were developed by adding 3,3′,5,5′-tetramethylbenzidine peroxidase substrate (KPL, Gaithersburg, MD). Reactions was stopped with 1M H_3_PO_4_ and analyzed at 450 nm by a Thermo ELISA plate reader.

### Monoclonal antibody affinity

The affinity constant (K_aff_) of the antibody was measured by ELISA, based on the Law of Mass Action, utilizing the OD_50_ of the sigmoid ELISA curve [29]. In this study, 96-well plates were coated with 200 ng and 100 ng of peptide corresponding to each antibody epitope. [Ab]_t_ and [Ab']_t_ represent the total antibody concentrations (M) in the wells at OD_50_ and OD_50_' for plates coated with [Ag] and [Ag'], respectively. K_aff_ (M^-1^) was calculated using the following equation: For [Ag'] = [Ag]/2, K_aff_ = 1/2(2[Ab']_t_−[Ab]_t_).

### *In vitro* virus binding assay

3 × 10^4^ HEp-2 cells were detached with 1 mM EDTA in PBS and incubated with purified RSV A2 particles for 30 min at 4°C. Then, the samples were blocked with anti-CD16/32 antibody, incubated with 1 μg of 5H6 or 3A5 for 30 min at 4°C, and stained with PE-conjugated rat anti-mouse IgG_1_. Finally, the cells were treated with DAPI solution to distinguish live cells. Flow cytometry was performed on LSR Fortessa (BD Biosciences) and the data was analyzed by using FlowJo software (Tree Star Inc.).

### Western blotting

Binding of mAb to wild-type Gcf and mutant Gcf was determined by a standard Western blotting. For 5H6 binding, 100 ng of proteins were processed with or without DTT. For 3A5 binding, 3 μg of proteins were separated using the same method. The gel was transferred to a PVDF membrane (Pall, Ann Arbor, MI, USA). The membrane was blocked and incubated with 5H6 and 3A5 from hybridoma for 2 hr at RT. After washing, the membrane was incubated with HRP-conjugated rabbit anti-mouse IgG.

### *In vitro* plaque reduction assay

200–300 PFU of RSV A2 were mixed with 2 μg of Palivizumab, heparin, 5H6, or 3A5 for 1 hr at 37°C. The mixture was then incubated with HEp-2 cells in 6-well plates for 1.5 hr at 37°C. After removing the inoculums, the cells were overlaid with 1% low melting agarose, and the plaques were counted 5 days later.

### Animals

Female BALB/c mice aged 6 weeks were purchased from Charles River Laboratories Inc. (Yokohama, Japan) and maintained under specific pathogen-free conditions at Ewha Womans University. All mice experiments were approved by Ewha Womans University Institutional Animal Care and Use Committee (Approval No. 14–083). For prophylactic mAbs injection, mice were administered intramuscular injections with 100 μg of each mAb in 100 μl of PBS. One day later, the mice were lightly anesthetized by isoflurane inhalation and challenged intranasally with 10^6^ PFU of RSV A2 strain in 50 μl of PBS (n = 4 mice/group). For vaccine-enhanced disease study, BALB/c mice were scarified with 6 × 10^6^ PFU of recombinant vaccinia virus expressing full length RSV G protein (vvG) using a 22-gauge needle. After 4 weeks, mice were intramuscularly administered with 300 μg of each mAb. All groups were challenged intranasally with 3 × 10^6^ PFU of RSV A2 1 days later (n = 4 mice/group) and then weighed each day.

### RSV titers in lung tissue

On day 4 post-infection, mice were sacrificed, and lung tissues were perfused and harvested. Lungs were passed through a 70 μm cell strainer into serum-free MEM. RSV titers in the lung supernatants were measured by standard plaque assay on HEp-2 cells. The data are expressed as PFU per gram of lung tissue. The limit of detection for RSV was 94 PFU/gram of lung tissue.

### *In vitro* chemotaxis assay

The *in vitro* chemotaxis assay was performed using a transwell insert plate with an 8 μm pore size (SPL). 10 μg of wild-type Gcf and 100 μg of mAbs (5H6 or 3A5) were mixed for 30 min at 4°C, and the mixture was added to the lower chamber of the insert plate. As a negative control, serum-free media alone or serum-free media that contained antibody (5H6 or 3A5) was added to the lower chamber. As a positive control, serum-free media that contained 10 μg of wild-type Gcf or media with 10% FBS was added to the lower chamber. 5 × 10^5^ THP-1 cells (ATCC, Manassas, VA) expressing CX3CR1 receptor were added to the upper chamber of the plate. The assembled plates were incubated for 5 hr at 37°C. Cells migrated to the lower chamber were counted, and the percent migration was determined using the following formula: 100 × {[(average number of migrated cells/area of the microscope viewing field) × area of the transwell insert]/number of cells seeded}.

### Statistical methods

Statistical analyses were performed using an unpaired, two-tailed Student’s t-test. The difference was considered statistically significant when the p value was ≤ 0.05.

## Results

### Generation and characterization of monoclonal antibodies against G core fragment

**Fig 1:**
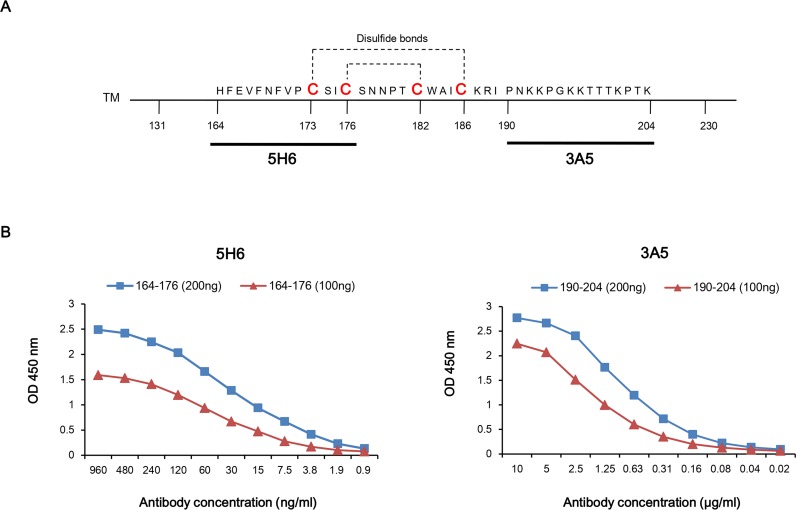
Epitope mapping and affinity measurement of two monoclonal antibodies (5H6, 3A5) against G core fragment. (A) Primary structure of G surface glycoprotein of RSV A2 strain and Gcf sequence. (B) Epitope mapping and affinity measurement of mAb (5H6 and 3A5) was performed using ELISA with known B-cell epitopes of the RSV G protein (peptide G/144-159, G/164-176, G/174-187, and G/190-204). The 96-well plates were coated with 200 ng or 100 ng of peptide corresponding to each epitope of the antibody. The wells were incubated with the indicated dilutions of mAbs.

Mouse monoclonal antibodies were generated by injecting highly purified recombinant G core fragment (Gcf) [20]. Two mAb clones, named 5H6 and 3A5, that showed high affinity to Gcf were selected and purified from hybridoma culture. To investigate the epitopes of these mAbs, peptides that correspond to known B-cell epitopes within Gcf were synthesized (peptide G/144-159, G/164-176, G/174-187, and G/190-204) [28]. ELISA results showed that 5H6 clone specifically binds to the G/164-176 peptide, which includes conserved sequences among RSV strains, while 3A5 clone binds to the G/190-204 peptide (Fig 1A).

ELISA was then performed to measure the affinity constant (*K*_aff_) of the antibody. The concentration of 5H6 at OD_50_ when 200 ng or 100 ng of peptides were coated was 30 ng/ml and 42 ng/ml, respectively. The concentration of 3A5 at OD_50_ when 200 ng or 100 ng of peptides were coated was 0.68 μg/ml and 1.12 μg/ml, respectively (Fig 1B). The *K*_aff_ (M^-1^) value for 5H6 was 1.66 × 10^9^, and that for 3A5 was 5.6 × 10^7^, indicating that 5H6 binds to the epitope with 30-fold higher affinity than 3A5 does.

**Fig 2:**
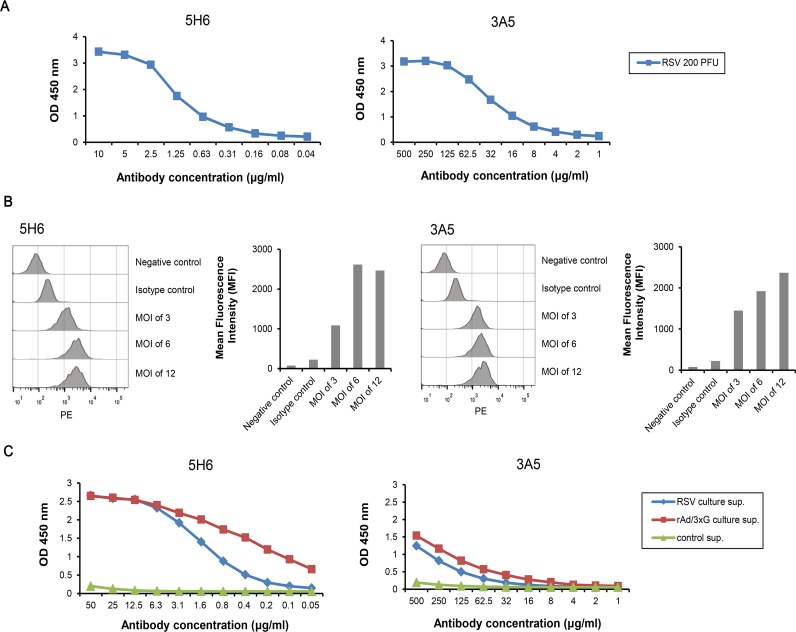
Binding characteristics of monoclonal antibody to G proteins expressed by mammalian cells. (A) 96-well plates were coated with 200 PFU of purified RSV A2 virus particles. 5H6 was serially diluted from 10 μg/ml, and 3A5 from 500 μg/ml. The analysis was performed by using ELISA. (B) *In vitro* binding assay using flow cytometry. Binding of 5H6 or 3A5 to RSV particles was indicated by histograms and the mean fluorescence intensity (MFI). (C) Secreted G protein and Gcf were prepared from HEp-2 cells infected with RSV A2 and rAd/3XG, respectively. 96-well plates were coated with 5 μg of supernatant per well.

We next investigated the binding characteristics of mAbs to G protein and derivatives from various sources. To assess mAbs binding to RSV particles, ELISA was performed with purified RSV A2 virus particles as coating antigen. The antibody concentration of 5H6 and 3A5 binding to RSV particles at OD_50_ was 0.8 μg/ml and 20 μg/ml, respectively, suggesting that 5H6 binds with 25-fold higher affinity than 3A5 does (Fig 2A). To confirm binding of mAbs to RSV particles, *in vitro* virus binding assay was performed using flow cytometry. 5H6 or 3A5 treated cells resulted in increases in the specific fluorescent signal compared to untreated cells or isotype control (Fig 2B). To test if mAbs bind to the native soluble G protein and/or Gcf expressed by mammalian cells, HEp-2 cells were infected with RSV A2 or recombinant adenovirus expressing tandem repeated copies of Gcf (rAd/3XG; [30]), and the supernatant from each culture was analyzed using ELISA. The results suggest that 5H6 binds to the mammalian-expressed G protein and Gcf in the RSV or recombinant adenovirus-infected culture supernatant at high affinity, respectively, whereas 3A5 binds at much lower levels (Fig 2C).

**Fig 3:**
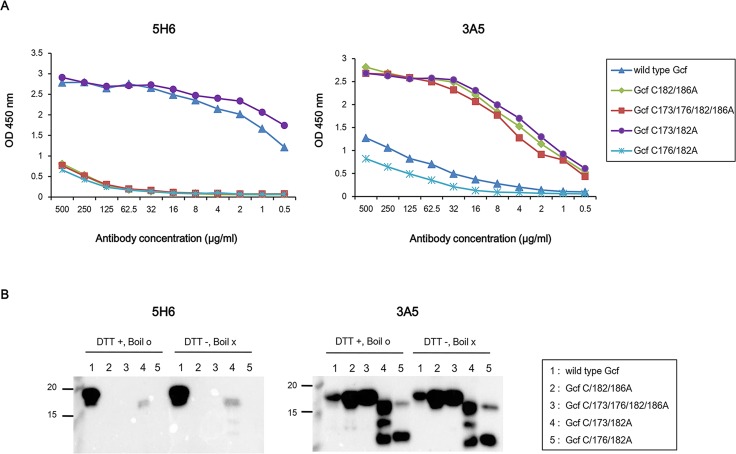
Binding characteristics of monoclonal antibodies to Gcf derivatives with cysteine substitutions. (A) 96-well plates were coated with 50 ng of Gcf and mutant Gcf derivatives per well. Antibodies were serially diluted from 500 μg/ml and ELISA was performed. (B) Western blotting analysis with Gcf and mutant Gcf derivatives. For this, 100 ng and 3 μg of each indicated protein were loaded for blotting with 5H6 and 3A5, respectively.

We next investigated if conserved cysteine residues are critical to the binding of mAbs using mutant Gcf derivatives in which cysteine residues were substituted with alanine. Binding of mAbs to wild-type Gcf and its derivatives was examined by both ELISA and Western blotting. ELISA results indicated that 5H6 specifically binds to wild-type Gcf and Gcf C173/182A mutant only. Interestingly, 5H6 binds to wild-type Gcf but does not bind to the other Cys mutants in western blotting analysis. However, 3A5 binds to Gcf C173/182A, C182/186A, and C173/176/182/186A but relatively weakly to wild-type Gcf and mutant Gcf C176/182A, in western blotting as well as ELISA (Fig 3A and 3B). These results suggest that Cys-176 and Cys-186 residues are essential for 5H6 binding to G protein.

### *In vitro* Neutralization and *In vivo* RSV clearance activity of 5H6 and 3A5

**Fig 4:**
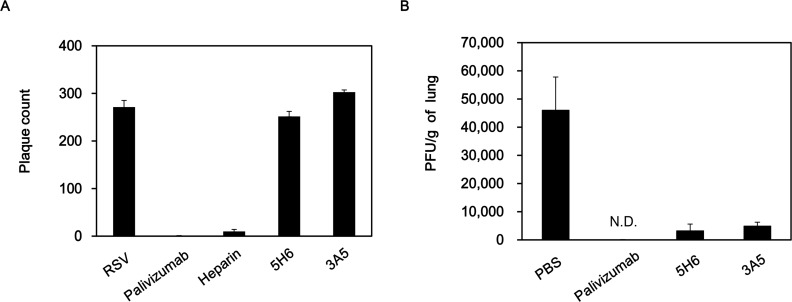
*In vitro* Neutralization and *In vivo* RSV clearance activity of 5H6 and 3A5. (A) *In vitro* plaque reduction assay. Palivizumab, heparin, 5H6, and 3A5 were incubated with 200–300 PFU of RSV A2 for 1 hr at 37°C. Then, the mixture was added to HEp-2 cells for 1.5 hr, and the plaques were counted 5 days later. (B) Prophylactic treatment with mAb in mouse model. BALB/c mice were intramuscularly administered 100 μg of each mAb and challenged intranasally with 10^6^ PFU of RSV A2 1 day later (n = 4 mice/group). On day 4 post-infection, lung homogenates were prepared, and lung viral titers were measured by plaque assay. The limit of detection was 94 PFU/gram of lung tissue. N.D., not detected.

In the previous results, we confirmed that 5H6 and 3A5 mAbs bind RSV particles and mammalian cell-expressed G proteins. To assess *in vitro* neutralization activity, RSV was incubated with Palivizumab, heparin, 5H6, and 3A5 prior to the infection on HEp-2 cells. The G protein contains heparin binding domains and thus heparin inhibits the binding of RSV to HEp-2 cells [31]. As shown in Fig 4A, Palivizumab treatment reduced the plaque by ~ 99.7%, while heparin treatment exhibited ~ 96% reduction. However, 5H6 and 3A5 did not show any effect on plaque reduction *in vitro* (Fig 4A). These results indicate that 5H6 and 3A5 do not have neutralizing activity against RSV *in vitro*.

To investigate whether 5H6 and 3A5 contribute to viral clearance in the lung, BALB/c mice were intramuscularly administered each mAb 24 hr before RSV A2 infection and viral titers in the lungs were determined 4 days postinfection. Viral titers were significantly reduced by 5H6 and 3A5 injection (~93% and ~90%, respectively; Fig 4B). No detectable RSV plaque was observed in the lungs of mice treated with Palivizumab. These results indicate that injection of these mAbs contributes to RSV clearance *in vivo*.

### Inhibition of Gcf-associated chemotactic activity by 5H6 and 3A5

**Fig 5:**
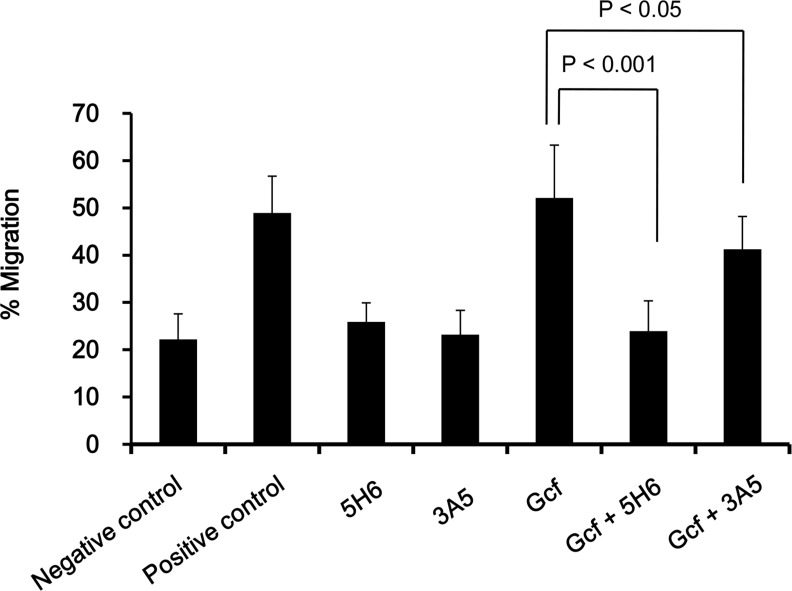
Inhibition of Gcf-associated chemotactic activity by 5H6 and 3A5. Inhibition of *in vitro* chemotaxis activity of Gcf by mAbs was analyzed using chemotaxis assay with THP-1 cells. 10 μg of wild-type Gcf were pre-incubated with 100 μg of mAbs in the lower chamber, and 5 × 10^5^ THP-1 cells were added to the upper chamber later. Serum-free media alone or media with 10% FBS was used as a negative or positive control, respectively. The assembled plates were incubated at 37°C for 5 hr. Cells attached to bottom of the membrane were counted, and the percent migration was calculated.

We have previously shown that wild-type Gcf displays chemotactic activity in an *in vitro* chemotaxis assay [20]. Thus, we examined whether 5H6 and 3A5 treatment inhibit the chemotactic activity of Gcf *in vitro*. When wild-type Gcf and 5H6 mAb were pre-incubated, migration of THP-1 cells were significantly decreased (p < 0.001) when compared to the controls. In contrast, 3A5 treatment decreased the migration significantly but less efficiently than 5H6 (p < 0.05) (Fig 5). These results indicate that 5H6 and 3A5 mAbs actively inhibit G protein-associated chemotactic activity in vitro.

### 5H6 prevents vaccine-enhanced weight loss

**Fig 6:**
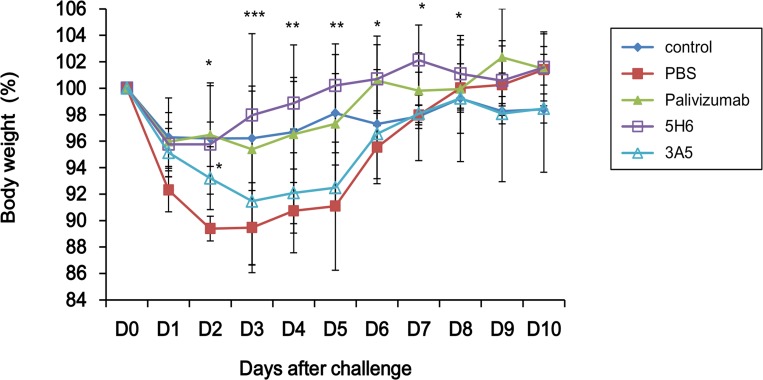
5H6 prevents weight loss primed by vvG. Effect of prophylactic administration of mAb 5H6 and 3A5 on weight change in BALB/c mice. BALB/c mice were scarified with 6×10^6^ PFU of recombinant vaccinia virus expressing RSV G protein (vvG). After 4 weeks, mice were intramuscularly administered with 300 μg of each mAb. All groups were challenged intranasally with 3 × 10^6^ PFU of RSV A2 1 day later (n = 4 mice/group) and then weighed each day. *, p < 0.05; **, p < 0.01; ***, p < 0.001, Significant increase on body weight for 5H6 or 3A5 treated group compared to PBS group.

It has been demonstrated that immunization with recombinant vaccinia virus expressing full length RSV G protein (vvG) results in vaccine-enhanced diseases including weight loss, pulmonary eosinophilia and lung immunopathology after a subsequent RSV challenge [32–35]. We determined if prophylactic injection of 5H6 and 3A5 could prevent vaccine-enhanced disease such as weight loss in vvG priming model. BALB/c mice were immunized with vvG and intramuscularly administered with each mAb 4 weeks after immunization. From day 2 to day 5 post RSV infection, the weight loss for 5H6-treated mice were significantly reduced (p < 0.001) comparable to the positive control Palivizumab group (Fig 6). In contrast, the weight recovered in 3A5-treated mice were significant only on day 2 post infection (p < 0.05) compared to the PBS negative control group. As a positive control, the Palivizumab group showed no significant weight loss until day 10. These results demonstrate that prophylactic injection of 5H6 could prevent vaccine-enhanced diseases such as weight loss induced by vvG immunization.

## Discussion

In this study, we characterized two mAbs against RSV G protein. The G protein plays a role in host cell attachment [36]. Additionally, the G protein has a CX3C motif that mimics fractalkine, competing for attachment to CX3CR1+ cells [15, 21]. Thus, the G protein-CX3CR1 interaction causes reduced migration of CX3CR1+ immune cells to infected lungs and downregulation of Th1-mediated immune response, thereby enhancing the Th2-biased immune responses [23–25]. Because the G protein is thought to be an important contributor to RSV disease pathogenesis, it might be a potential target for vaccines and therapeutic antibodies. Our results demonstrated that prophylactic injection of mAbs 5H6 and 3A5 promotes viral clearance in the lungs (Fig 4B). It has been previously reported that mice treated intraperitoneally with 300 μg of anti-RSV G protein mAb 131-2G against amino acids 164–176 showed 99% reduction in viral titers in the lung [37]. In an another study, mice treated intravenously with 131 μg of anti-RSV G protein mAb 18A2B2 against amino acids 174–187 were protected from RSV infection [38]. In our study, 5H6 and 3A5 did not inhibit RSV infection *in vitro*, even at concentrations of 100–200 μg (Fig 4A). There are several possible mechanisms for the difference in viral clearance activity of mAbs *in vivo* and *in vitro*. First, mAb might increase of RSV clearance through Fc- or complement-mediated mechanisms *in vivo*. When antigen-antibody complexes (immune complexes) are formed, the phagocytes bind to the Fc region of IgG and then initiate phagocytosis of immune complexes. Additionally, complement binding to the Fc region of immune complexes facilitates opsonization. Another possible mechanism is that antibody-dependent cellular cytotoxicity (ADCC) is involved in virus clearance activity *in vivo*. It has been demonstrated that mAb 131-2G injection resulted in the increase of RSV clearance even though it does not neutralize RSV *in vitro* [37, 39]. This effect requires Fc region since prophylaxis with the F(ab′)_2_ form of 131-2G did not decrease pulmonary virus replication [37]. Similarly, treatment with mAb 18A2B2 in complement-deficient mice or treatment with mAb F(ab′)_2_ fragments did not protect against RSV infection [38].

Our study also demonstrated that 5H6 and 3A5 inhibit Gcf-associated chemotactic activity *in vitro* (Fig 5). We had previously shown that wild-type Gcf displays chemotactic activity and conserved four cysteine residues are necessary for Gcf-associated chemotactic activity [20]. When Gcf was mixed with mAbs, the number of migrated THP-1 cells was reduced by 5H6 (p < 0.001) and 3A5 (p < 0.05). Although 5H6 binds to G/164-176, and 3A5 binds to G/190-204, these mAbs might shield the CX3C motif that spans amino acids 182–186.

Our study demonstrated that prophylactic injection of 5H6 prevented weight loss induced by vvG priming and subsequent RSV infection (Fig 6). We have suggested that 5H6 structurally shields the CX3C motif within the Gcf and inhibits Gcf-associated chemotactic activity *in vitro*. Therefore, it seems that 5H6 inhibits the binding of CX3CR1+ cells to CX3C motif within the G protein. CX3CR1 is generally expressed on CD8 T cell, CD4 Th1 cell, NK cell and monocytes/macropahges [40, 41]. It has been demonstrated that RSV G protein CX3C motif reduced CX3CR1+ cell trafficking to the lungs during infection and eventually decreased antiviral response to RSV infection [23]. Also, 131-2G-treated, RSV-challenged mice showed reduction in disease indicators including weight loss, and increased Th1-associated cytokines [42]. Thus, we hypothesize that trafficking of Th1 cells to the lungs is increased and excessive Th2-mediated immune responses that induce the pathology might be suppressed by 5H6.

The amino acids 164–176 and Cys-186 of the G protein are required in order for mAb 131-2G to bind. However, Western blotting showed that 131-2G did not bind with the recombinant RSV that had only a C186S mutation of the G protein [39, 43]. To investigate whether binding of mAbs correlates with conserved Gcf cysteine residues, we generated mutant Gcf proteins in which cysteine residues were substituted with alanine. 5H6 binds well to wild-type Gcf that has four conserved cysteines, but does not bind mutant Gcf C182/186A that has amino acids 164–176 (Cys-173, 176) but lacks Cys-186 (Fig 3A and 3B). Therefore, similar to 131-2G, Cys-186 is required for 5H6 binding. Also, mutant Gcf C173/182A that has Cys-176 and 186 is recognized by 5H6, compared to mutant Gcf C176/182A that has Cys-173 and 186. This result indicates that Cys-176 is more important than Cys-173 for 5H6 binding.

In conclusion, we demonstrated that mAbs against RSV G protein contribute to RSV clearance *in vivo* and reduce vaccine-enhanced weight loss. Therefore, these antibodies could be used as a prophylactic regimen against RSV infection.
